# Reducing the dosing frequency of selective digestive tract decontamination to three times daily provides effective decontamination of Gram-negative bacteria

**DOI:** 10.1007/s10096-021-04234-1

**Published:** 2021-04-01

**Authors:** Jara R. de la Court, Kim C. E. Sigaloff, Thomas Groot, Johan I. van der Spoel, Rogier P. Schade

**Affiliations:** 1 Department of Medical Microbiology and Infection Prevention, Amsterdam University Medical Center, Room ZH 3A74, de Boelelaan 1117, 1081 HV Amsterdam, The Netherlands; 2 Department of Infectious Diseases, Amsterdam University Medical Center, Amsterdam, The Netherlands; 3 Department of Intensive Care Medicine, Amsterdam University Medical Center, Amsterdam, The Netherlands

**Keywords:** Selective decontamination, Antibacterial agents, Antimicrobial stewardship, Infection prevention, Intensive care unit

## Abstract

This study evaluated the effectiveness of selective digestive tract decontamination (SDD) application three times daily (t.i.d.) compared to the standard four times daily (q.i.d.). Retrospective equivalence (combined non-inferiority and non-superiority design) study with a before-and-after design on a tertiary ICU in which the SDD frequency was reduced from q.i.d. to t.i.d. All patients with ICU admissions ≥72h and with ≥2 surveillance cultures collected on different dates were included in this study. We compared successful decontamination of Gram-negative bacteria (GNB). Furthermore, time to decontamination, ICU-acquired GNB bacteraemia and 28-day mortality were compared between the two groups. In total 1958 ICU admissions (1236 q.i.d., 722 t.i.d). Decontamination was achieved during the first week of admission in 77% and 76% of patients receiving SDD q.i.d and t.i.d., respectively. Successful decontamination within 14 days (without consecutive acquisition of Gram-negative bacteria) was achieved in 69.3% of the admissions with q.i.d. versus 66.8% in t.i.d. SDD (*p*-value = 0.2519). The proportions of successful decontamination of GNB were equivalent in both groups (−0.025, 98% CI: −0.087; 0.037). There was no significant difference in time to decontamination between the two regimens (log-rank test *p*-value = 0.55). Incidence (episodes/1000 days) of ICU-acquired GNB bacteraemia was 0.9 in both groups, and OR for death at day 28 in the t.i.d. group compared to the q.i.d. group was 0.99 (95% confidence interval, 0.80–1.21). This study shows that a t.i.d. application regimen achieves similar outcomes to the standard q.i.d. regime, for both microbiological and clinical outcome measures.

**Supplementary Information:**

The online version contains supplementary material available at 10.1007/s10096-021-04234-1.

## Introduction

Infection is a major complication among patients in the intensive care unit (ICU) resulting in additional morbidity, higher risk of mortality and increasing health care costs [1]. Selective digestive tract decontamination (SDD) is a common measure to prevent infections on the ICU. The principle behind SDD is that by reducing the numbers of potentially pathogenic microorganisms (PPMs) in the gut, the risk of ICU-acquired infections can be reduced [2]. Intestinal decontamination of Gram-negative bacteria (GNB) was associated with a threefold reduction in ICU-acquired bacteraemia with GNB [3]. Four cluster-randomized controlled trials have previously investigated the efficacy of SDD, the positive effect of SDD on clinical outcomes, i.e. improved survival and less infectious complications while maintaining a low prevalence of antibiotic resistance, has been demonstrated by three cluster-randomized studies [3–6].

The SDD regime is applied in ventilated patients with an expected duration of artificial ventilation > 48 h and consists of a mixture of non-absorbable antimicrobials combined with intravenous cefotaxime during the first 4 days of ICU admission. SDD is applied q.i.d. and this frequency has remained unchanged since the first SDD studies in the 1980s. There have been no studies that evaluated the optimal dosing frequency of SDD in order to achieve GNB decontamination. Reducing the dosing frequency of SDD paste application from four to three times daily (t.i.d.) would lower antimicrobial consumption and related health care costs. More importantly, by reducing the dosing frequency, the nightly administration of SDD paste application could be omitted, thereby preventing sleep interruption. Facilitating uninterrupted sleep reduces the incidence of delirium and is essential for adequate immune, metabolic and endocrine functioning [7, 8]. In an attempt to increase the quality of sleep for ICU patients, the dosing frequency of SDD application was reduced from q.i.d. to t.i.d in 2017. This t.i.d. SDD dosing regimen has been the standard of care since. This provided us with the opportunity to perform a before-and after study comparing the success of decontamination between q.i.d. versus the t.i.d. SDD application frequency. The primary objective of this study is to examine if the success of and time to GNB decontamination of the digestive tract is equivalent between the old (q.i.d.) and the new (t.i.d.) regimen. The secondary outcomes are relevant clinical outcomes: all-cause 28-day mortality and incidence of ICU-acquired GNB bacteraemia. Finally, we want to examine the relationship between the time to GNB decontamination and the risk of ICU-acquired GNB bacteraemia.

## Materials and method

### Setting, design and population

We retrospectively studied electronic microbiology and patient data gathered in the period from November 2011 until July 2019 on the ICU of the Amsterdam University Medical Centre, location VU Medical Centre (VUmc), a 28-bed ICU in a 730-bed tertiary care centre in the Netherlands. Data were derived from an automated database combining laboratory data with pseudo-anonymous patient data from the electronic patient dossier (EPD). This database was constructed for antimicrobial stewardship and infection prevention purposes and consists of microbiological data, admission and discharge data, Medicine Administration Records, Surgical interventions and a small amount of patient data: sex, date of birth, date of death. Data visualization was performed using TIBCO® Spotfire®. The non-absorbable antibiotics in the SDD regimen consist of application of paste (colistin 2%, tobramycin 2%, amphoterin B 2%) in the oral cavity and of suspension (colistin 100 mg, tobramycin 80 mg, amphotericin 500 mg) via the nasogastric tube. This regimen was applied q.i.d. until 26-05-2017; thereafter, the same regimen was applied t.i.d. Patients admitted to the ICU also receive 4 days of intravenous cefotaxime q.i.d. 1 g; this practice did not change over the course of time.

All adult patients with an ICU admission of at least 72h and with at least 2 surveillance cultures drawn on two separate days were included in the analysis.

### Microbiological methods

Surveillance cultures were taken on admission to the ICU and thereafter once a week on Mondays for pharynx and anus, and on Mondays and Thursdays for sputum. All surveillance cultures were included in the analysis. Surveillance cultures within 72h of admission represented the flora at admission on the ICU and are further called baseline surveillance cultures.

Based on previous literature, the following aerobic GNB were defined as PPMs: *Klebsiella*, *Enterobacter*, *Citrobacter*, *Proteus*, *Morganella*, *Serratia*, *Acinetobacter* and *Pseudomonas* species [9]. By assessing the prevalence of GNB in the blood cultures of the patients in our cohort, we found that *Stenotrophomonas maltophilia* was also a frequent cultured pathogen in the study period and was therefore added as an PPM. Because we also want to assess the reduction of carriage of endogenous “normal” but potentially pathogenic flora, we added *Escherichia coli* to the list.

In the Amsterdam UMC medical microbiology laboratories, antimicrobial susceptibility was tested using automated systems, gradient tests and/or using the disk diffusion method.

Decontamination was defined as the reduction of Gram-negative bacterial load to a level at which surveillance cultures are negative (rectal or faeces, pharyngeal and sputum). The number of days in which decontamination should occur to be considered successful, i.e. adequate to reduce infectious complications and mortality, has not been previously defined. In the study of de Smet et al.—in which the relationship between SDD and reduction of mortality was confirmed—the frequency of GNB isolation from rectal swabs among patients receiving SDD was reduced from 56% at day 3 to 15% at day 14 [4]. The SDD regimen used in the study of de Smet et al. was identical to the q.i.d. regimen used in this study. Therefore, we chose to define successful decontamination as a surveillance culture result negative for GNB within 14 days without positive follow-up surveillance culture for GNB during ICU-admission; i.e. follow-up lasted until the moment of discharge from the ICU.

In case of new fever, two blood cultures were drawn. ICU-acquired bacteraemia or candidemia was defined as bacteraemia or candidemia occurring at least 48 h after ICU admission with growth of either GNB (Enterobacterales or glucose-nonfermenting Gram-negative rods), candida species or Gram-positive bacteria (GPB) without documented bacteraemia or candidemia with the same species in the first 48 h of ICU admission. Polymicrobial bacteraemia was defined when one or more microorganisms (from the same group; either GNB, GPB or Candida spp.) were isolated from one or more blood cultures, and clinical evidence suggested they had arisen from a common source and were part of the same episode. If the source was unknown, all positive blood cultures occurring within 48 h of each other are considered a single bacteraemia. Bacteraemia due to coagulase negative staphylococci (CNS) or other common skin bacteria was defined as the presence of the same organism in two separate blood culture sets. Twenty-eight-day all-cause mortality is defined as death for any cause within 28 days after the date of admission to the ICU.

Due to the before-after study design, we anticipated that time-dependent factors such as antimicrobial resistance could introduce bias. We described the combined prevalence of susceptibility for the components of SDD (tobramycin and colistin) in surveillance cultures at baseline.

### Analysis

Two groups were formed on the basis of the dosing frequency of SDD, q.i.d. versus t.i.d. Continuous variables are presented as median and interquartile range. Categorical variables are presented as percentages. For the difference in proportion of patients with successful decontamination of PPMs in both groups and for comparison of susceptibility of Gram-negative bacteria at baseline, a chi-square test was used. For the difference in time to decontamination of PPMs from surveillance cultures, a Kaplan-Meier curve was used. For equivalence testing, the two one-sided test (TOST) procedure was used. The largest clinically acceptable effect for which equivalence can be declared was a mean difference of 10%. The equivalence limit was set to 0.1 (d_E_ = 0.1). All data available was used; no formal sample size was calculated. To test the association of time to decontamination of GNB and ICU-acquired bacteraemia, a landmark analysis was used for patients in which surveillance cultures are still available at day 3, 6, 9, 12, and 14 after admission. Cox-regression was used to examine if there was an association between success of decontamination of GNB and ICU-acquired bacteraemia and if this association differed between treatment groups. Odds ratio was calculated to compare 28-day all-cause mortality between the two groups. The association of time to decontamination of GNB and 28-day all-cause mortality was analysed, using cox-regression, in a subgroup of patients with a minimum of 14 days of surveillance culture data. Data analysis was performed using R Statistical Software (version 3.6.1; R Foundation for Statistical Computing, Vienna, Austria).

## Results

**Fig. 1:**
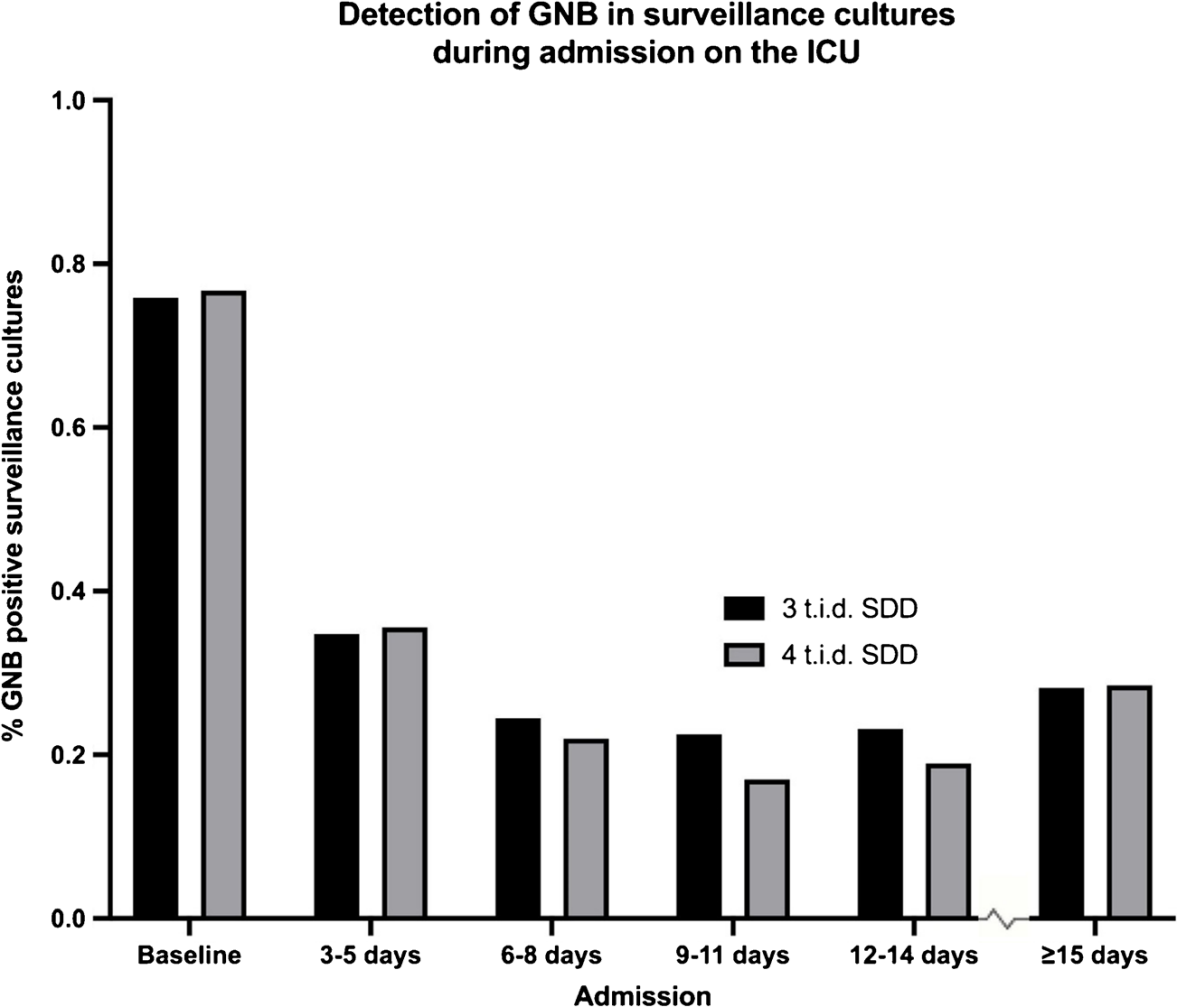
Percentages of patients with Gram-negative bacteria in surveillance cultures, out of all patients with surveillance cultures drawn during admission days, are presented

**Fig. 2:**
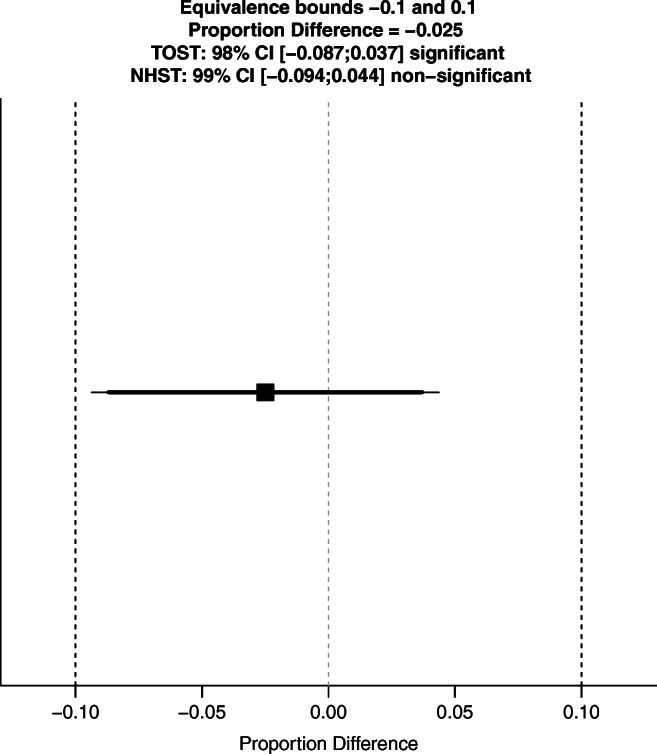
Equivalence test of proportion of successful decontamination

**Fig. 3:**
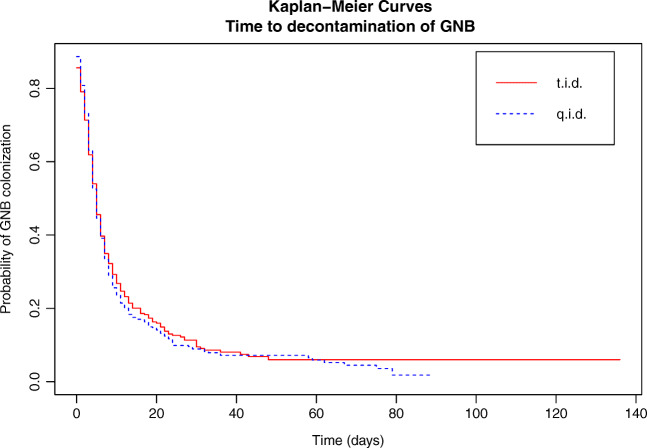
Time to decontamination of GNB (log rank *p*-value = 0.55)

Data was gathered from 3943 admissions, in 3662 ICU patients. A total of 1958 admissions in 1851 patients met the criteria for inclusion in our study. The q.i.d. cohort consisted of 1236 admissions, and the t.i.d. cohort consisted of 722 admissions. Baseline characteristics are shown in Table 1. The cohorts were comparable with regard to duration of admission, age at admission and percentage of male. Figure 1 shows, for both cohorts, the percentage of patients with positive cultures at the different time points during the admission. In both cohorts, successful decontamination was primarily initiated during the first week of admission, with 77–76% (q.i.d–t.i.d.) of patients with GNB positive cultures at admission to 22–24% (q.i.d–t.i.d.) after 1 week. The percentage of patients with GNB positive cultures varied between 17 and 24% during 6–14 days. However, after 15 days, rate of GNB positive cultures increased to 28% in both groups. Table 2 shows the proportion of successful decontamination in the two groups. Successful decontamination, defined as GNB negative surveillance cultures within 14 days without any further GNB detection in surveillance cultures thereafter, was not significantly different between the two groups (Table 2). To show non-inferiority of the t.i.d. regime, equivalence test of the proportions of successful decontamination was performed (Fig. 2). With an equivalence bound of 0.1 and a 98% confidence interval, the proportions of successful decontamination of GNB are equivalent in both groups. The time to decontamination of GNB is shown in Fig. 3. The log-rank test, to compare time to decontamination of GNB between the two cohorts, did not show any difference between the groups (*p*-value of 0.55).

**Table 1:** Baseline characteristics

	q.i.d. SDD	t.i.d. SDD
Total amount of admissions	1236	722
Total patients in cohort (% male)	1171 (67.3%)	690 (70%)
Admission duration in days; median (min–max)	11 (3–118)	11 (3–182)
Age at admission day; mean (min–max)	62 (17–92)	62 (17–92)

**Table 2:** Proportion of ICU admission with successful decontamination of GNB within 14 days (remaining negative during the entire admission period)

	q.i.d. SDD (%)	t.i.d. SDD (%)	Total
Failure of decontamination within 14 days	380 (30.7%)	240 (33.2%)	620 (31.7%)
Successful decontamination within 14 days	856 (69.3%)	482 (66.8%)	1338 (68.3%)
Total	1236	722	1958

We observed 27 episodes of ICU-acquired bacteraemia with GNB during the study period, 17/1236 before (1.4%) and 10/722 (1.4%) after adjustment of SDD application frequency, with an incidence of 0.9 episodes/1000 ICU days in both. Causative pathogens in intensive care unit–acquired bacteraemia are shown in Table 3.

**Table 3:** Causative pathogens in intensive care unit–acquired bacteraemia or candidemia (*n* = admission)

	q.i.d. SDD *n* (%)	t.i.d. SDD *n* (%)	Total *n* (%)
Gram-negative bacteraemia	17 (1.4)	10 (1.4)	27 (1.4)
*Pseudomonas aeruginosa*	4	3	7
*Escherichia coli*	3	3	6
*Enterobacter species*	3	0	3
*Klebsiella species*	2	0	2
*Stenotrophomonas maltophilia*	1	1	2
*Acinetobacter species*	0	1	1
*Moraxella osloensis*	1	0	1
*Ochrobactrum anthropi*	1	0	1
*Burkholderia cepacia*	0	1	1
Polymicrobial	1	1	2
Gram-positive bacteraemia	110 (8.9)	87 (12.0)	197 (10.1)
*Staphyloccocus aureus*	12	7	19
* Streptococci* spp.	0	3	3
CNS	34	24	58
*Enterococcus faecium*	37	18	55
*Enterococcus faecalis*	10	7	17
Polymicrobial*	17	28	45
Candidemia	11 (0.9)	4 (0.6)	15 (0.8)
*Candida albicans*	2	3	5
*Candida dubliniensis*	1	0	3
*Candida glabrata*	2	1	2
*Candida kefyr*	1	0	1
*Candida krusei*	2	0	1
*Candida lusitaniae*	1	0	1
*Candida parapsilosis*	1	0	1
*Candida tropicalis*	1	0	1
Total amount of admissions	1236	722	1958

*Four patients with more than one Gram-positive bacteraemia (defined as polymicrobial Gram-positive bacteraemia) had a *streptococci* spp. bacteraemia during their admission (2 during q.i.d., 2 during t.i.d.)

Successful decontamination of GNB within 3, 6, 9, 12, and 14 days all significantly reduced the HR (ranging from 0.107 to 0.279) of ICU-acquired bacteraemia with GNB (supplemental figures 1-5). This difference was not influenced by treatment group (supplemental figure 6). Successful decontamination of GNB was not associated with the occurrence of ICU-acquired bacteraemia with *S. aureus*, *Streptococci* spp. or with ICU-acquired candidemia (supplemental table 1). The proportions of ICU-acquired bacteraemia did not differ between the two regimens except for GPB. The increase in *Streptococci* spp. bacteraemia after reducing the dosing frequency to t.i.d. was not found to be significant (*p*-value = 0.0577). Twenty-eight-day all-cause mortality was 26.1% and 25.6% in the q.i.d. and t.i.d. groups; odds ratios for death at day 28 in the t.i.d. group compared to the q.i.d. group was 0.99 (95% confidence interval [CI], 0.80–1.21). We did not find an association between the success of decontamination and 28-day mortality (*p*= 0.593)

To control for potential changes in resistance epidemiology between the two time periods, especially with regard to the incidence of bacteria that were susceptible to the SDD antibiotics, we compared the surveillance cultures at baseline (Table 4). Between 72 and 71% (for the q.i.d. and t.i.d. group) of all admissions started with GNB positive surveillance cultures at baseline. Susceptibility for tobramycin or colistin in Gram-negative bacteria in baseline surveillance cultures was 97.3% and 96.8% (for the q.i.d. and t.i.d. group) with a *p*-value of 0.61 using the chi-square test. We conclude that the baseline epidemiology at admission on the ICU is comparable, and that our results are not biased by an epidemiological shift in susceptibility rate between the two historical cohorts.

**Table 4:** Susceptibility for tobramycin or colistin in Gram-negative bacteria in baseline surveillance cultures

	Admissions during q.i.d. SDD application period *n* (%)	Admissions during t.i.d. SDD application period *n* (%)
Total	1236	722
Gram-negative bacteria in baseline (<72 h) surveillance cultures	880 (71.2)	518 (71.7)
Susceptibility for at least one of the non-absorbable antibiotics in SDD (tobramycin or colistin) in Gram-negative bacteria in baseline (<72 h) surveillance cultures	852/880 (96.8)	504/518 (97.3)

The incidence of VAP on our ward has been shown to be 3.3/1000 ventilation days [10]. Prevalence measurements for the national ‘PREZIES’ survey of hospital infections are performed every 3 months [11, 12]. Based on these surveys, the prevalence of VAP in q.i.d. cohort (median 0.1% of admitted patients, range 0–19%) did not significantly increase after change to the t.i.d. regime (median 0.1% of admitted patients, range 0–21%).

## Discussion

The optimal SDD dosing regime has not previously been evaluated in a clinical setting. The present study demonstrated that a t.i.d. application regime application regimen provides equally effective digestive tract decontamination compared to the standard q.i.d. regime. SDD effectiveness was demonstrated within a large patient population (*n* = 1958) receiving either t.i.d. (*n* = 722) or q.i.d. (*n* = 1236) administration. Several outcome measures support our conclusion. First, the proportion of successful decontamination was equal in both groups. Second, the time to decontamination of GNB did not, at any time point, differ significantly. Finally, we found no significant differences in clinically relevant outcomes (i.e. ICU-acquired bacteraemia and 28-day all-cause mortality) between the two cohorts.

Although the goal of SDD is digestive tract decontamination and subsequent reduction of ICU-acquired infection, the four cluster-randomized controlled trials that have previously investigated the efficacy of SDD did not report the time to or success of decontamination [3–6]. The primary outcome of our study can therefore not directly be compared to these trials. However, the close association between gut (de-)colonization and ICU-acquired infection is well established and confirmed by our data [13–18]. Frencken et al. showed that both rectal and respiratory tract colonization were associated with bacteraemia (cause-specific hazard ratios, 7.37 [95% CI, 3.25–16.68] and 2.56 [95% CI, 1.09–6.03], respectively) [14]. Oostdijk et al. found that respiratory tract decolonization and intestinal tract decolonization were associated with a 33% and 45% reduction in the occurrence of intensive care unit–acquired Gram-negative bacteraemia, respectively. In our data, we showed that success of decontamination of GNB at different time points all reduced the risk of ICU-acquired bacteraemia with GNB; this reduction was most prominent in patients who were decontaminated within 6 days (HR = 0.107). Moreover, Oostdijk et al. reported a reduction of proportion of colonization in patients treated with SDD throughout intensive care unit stay from approximately 30% at day 1 to 15–20% at day 20 [13]. The fact that decontamination rates found in our study are comparable to the results found in the large prospective study of the Smet et al. (i.e. 85% of patients cultured after 14 days are decolonized from GNB), in which clinical effectiveness of SDD application was proven, is a clear indication that t.i.d. administration of SDD is clinically effective and safe [4, 13]. Furthermore, the secondary clinical outcomes defined in our study (ICU-acquired bacteraemia with GNB and 28-day all-cause mortality), in which t.i.d. proved to be non-inferior to q.i.d, supports this conclusion.

No significant differences were found in the incidence of ICU-acquired bacteraemia (*S*. *aureus*, *Streptococci *spp., candidemia and GNB) between the two regimes. Furthermore, VAP incidence (3.3/1000 ventilation days) on our ward is low compared to other European centres in which incidence density of 18.3/1000 ventilation days was reported [19]. The prevalence of blood stream infections of any pathogen, excluding CNS, *Micrococcus*, and *Clostridium* species and non-pneumococcal *Streptococci*, was (123/2082) 5.9% in the SDD study group by van Wittenkamp et al. [6], which is comparable to the prevalence (6.8%) found in our study, excluding all CNS, polymicrobial GPB and (non-pneumococcal) *Streptococci* bacteraemias. Pseudomonas bacteraemias are of great concern; however, the incidence of pseudomonas bacteraemias in our study is low compared to benchmarking incidences (0.4% in this study compared to 0.7% found by Hurley et al) [20].

Strengths of our study are the size of our study population and the detailed information about intestinal colonization during SDD. This study provides, for the first time, detailed insight into the underlying dynamics of culture results during SDD. We demonstrated equal microbiological and clinical effectiveness of less frequent dosing. This is reflected in a stable incidence of ICU-acquired GNB bacteraemia and 28-day all-cause mortality. Our study also has limitations. We used a monocentric retrospective approach, using a historical control group. We had no detailed clinical information on ventilator-associated pneumonias in individual patients. On a population level, however, we noted no significant change in prevalence of VAP since the introduction of the t.i.d. application regime [11]. Besides, Bergmans et al. showed previously that decolonization of the respiratory tract results in a relative risk reduction of 67% in the incidence of VAP. This makes a difference in VAP incidence despite the equivalent decontamination rates in our cohorts unlikely [21]. The retrospective design precludes correction for hidden variables in the original data that might have confounded the results. Yet, potential confounders in the original uncontrolled data are likely to be present in both groups (t.i.d. versus q.i.d.). Specifically, we ruled out an epidemiological shift in susceptibility for the SDD antibiotics, which could otherwise have biased the results.

Our findings support a t.i.d. SDD application frequency in the ICU. This new regime was designed as a sleep-promoting intervention on the ICU. Many sleep-disturbing factors are present on the ICU, but clinical interventions are one of the most important disruptive factors and should therefore be avoided [22]. Furthermore, any unnecessary antibiotic use should be avoided to reduce the harm that can result from antibiotic-associated adverse events [23]. During the t.i.d. SDD application period of 3 years tobramycin and colistin resistance did not change, which is in line with previous studies assessing antibiotic resistance during the use of SDD [24–26].

## Conclusion

Based on time to and success of decontamination of Gram-negative bacteria, incidence of ICU-acquired GNB bacteraemia (0.9/1000 ICU days) and 28-day all-cause mortality, there is no difference between a t.i.d. and a q.i.d. SDD application regime. These study findings justify implementation of a t.i.d. SDD application regimen in ICUs where a standard (q.i.d.) regimen is in place.

## Supplementary Information

ESM 1 (DOCX 670 kb)

## Data Availability

Data is available upon request.
