# Supplementary material for: Reducing the dosing frequency of selective digestive tract decontamination to three times daily provides effective decontamination of Gram-negative bacteria
Source: Eur J Clin Microbiol Infect Dis. 2021 Apr 1;40(9):1843–50. doi: 10.1007/s10096-021-04234-1 (PMC8012068; doi:10.1007/s10096-021-04234-1)
Supplement: Supplementary file 1 — (DOCX 670 kb) [file 10096_2021_4234_MOESM1_ESM.docx]

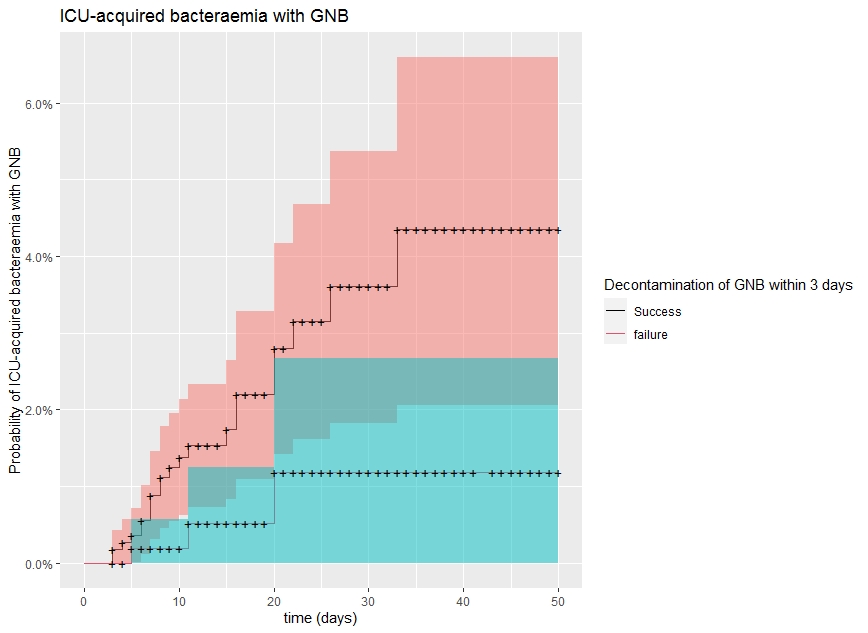
Figure 1. Probability of ICU-acquired bacteraemia with GNB, comparing success of decontamination of GNB within 3 days, p-value = 0.039, HR = 0.279


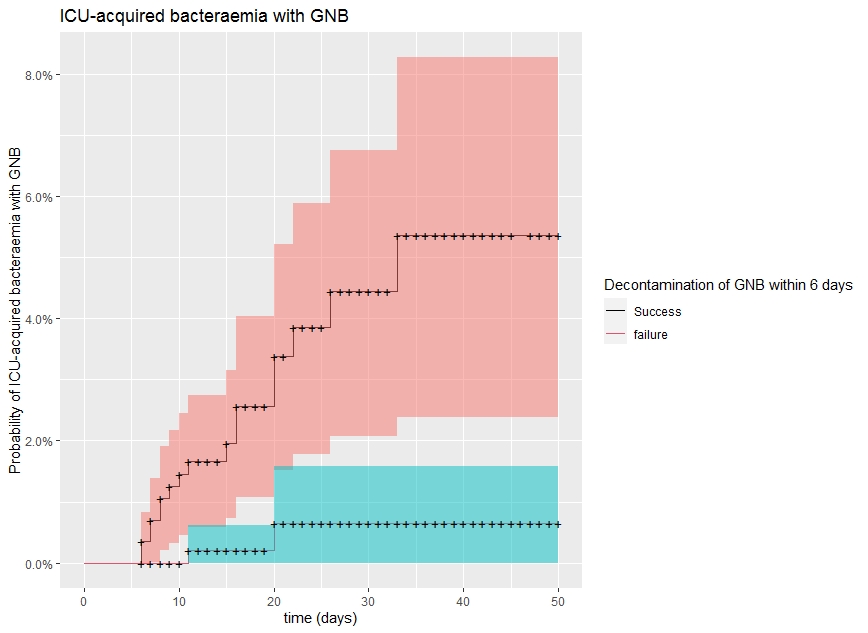
Figure 2. Probability of ICU-acquired bacteraemia with GNB, comparing success of decontamination of GNB within 6 days, p-value = 0.003, HR = 0.107


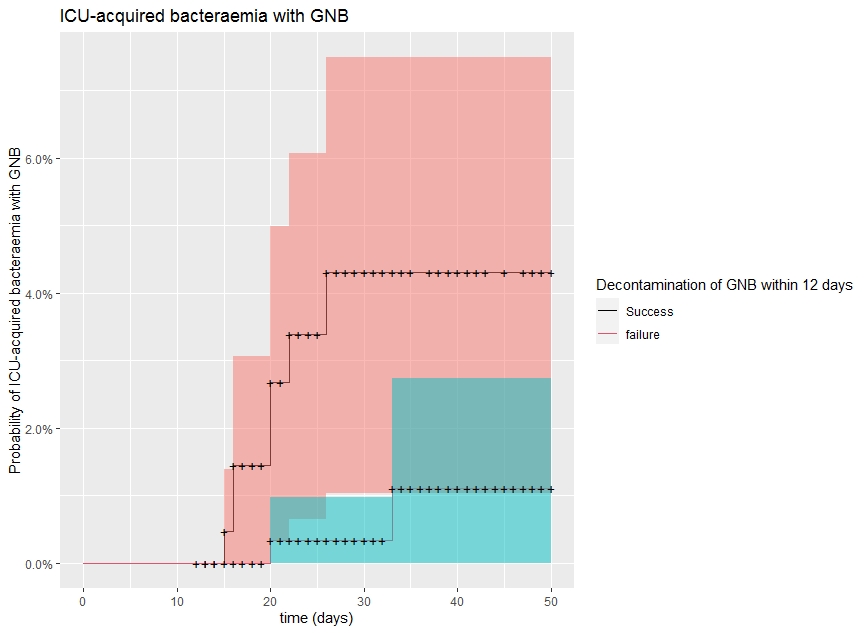
Figure 4. Probability of ICU-acquired bacteraemia with GNB, comparing success of decontamination of GNB within 12 days, p-value = 0.019, HR = 0.151


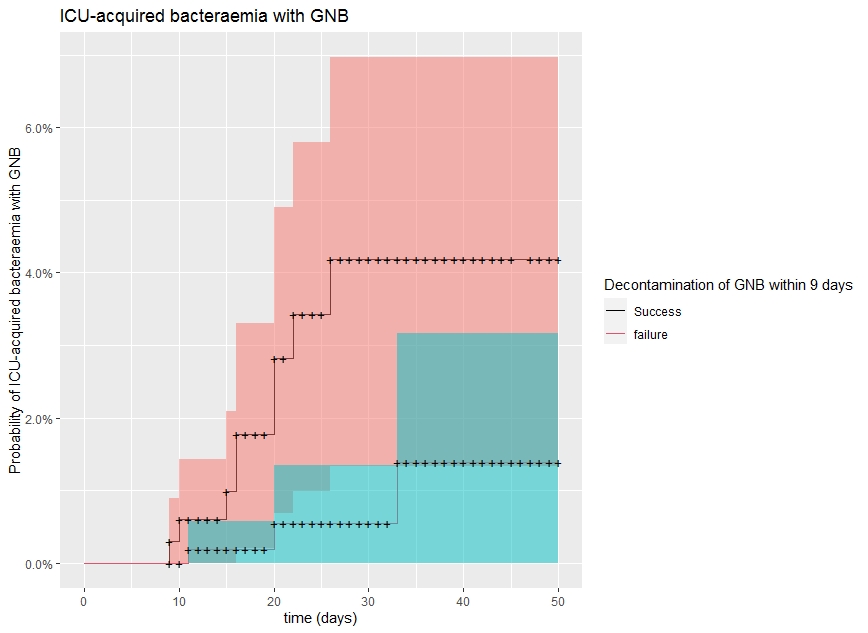
Figure 3. Probability of ICU-acquired bacteraemia with GNB, comparing success of decontamination of GNB within 9 days, p-value = 0.037, HR = 0.285


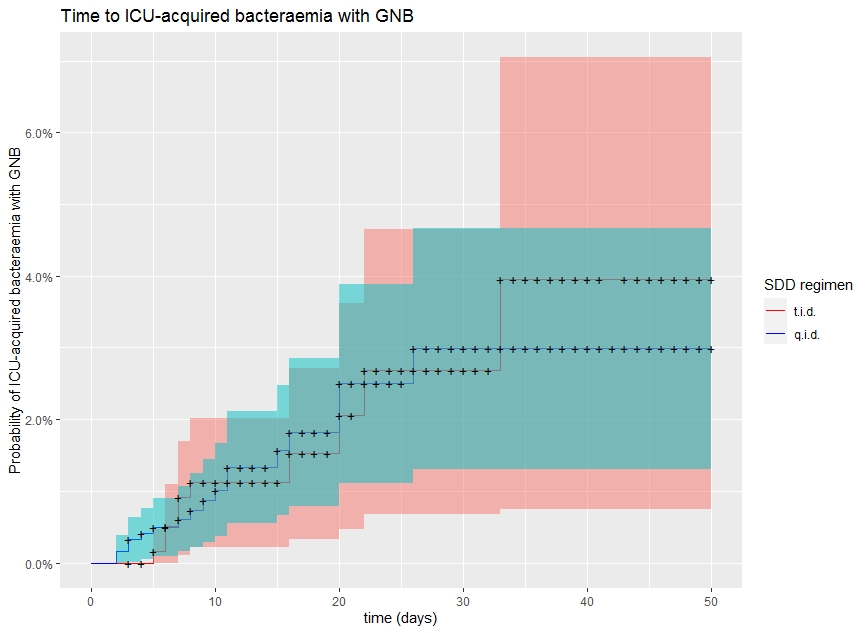


Figure 6. Probability of ICU-acquired bacteraemia with GNB, comparing q.i.d. and t.i.d., p-value = 0.98, HR = 1.01


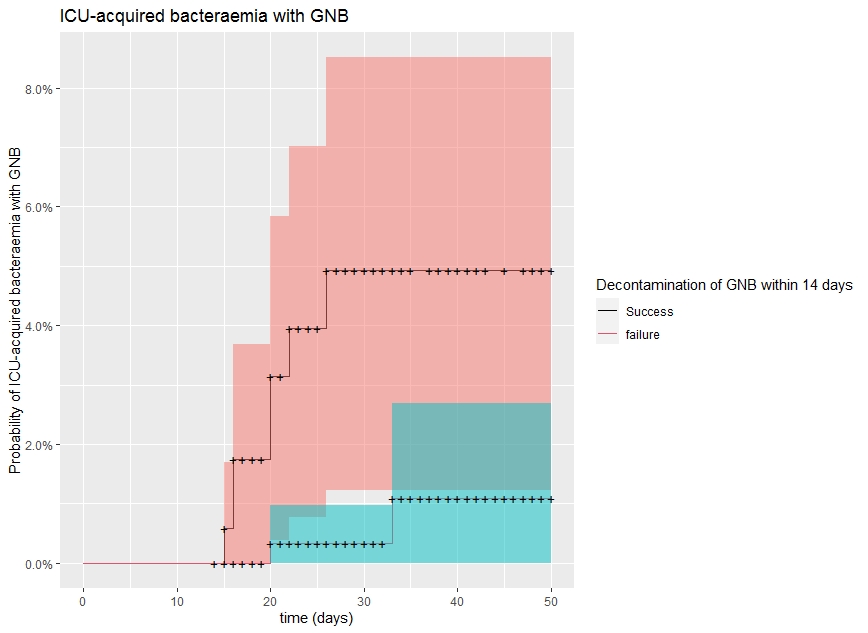
Figure 5. Probability of ICU-acquired bacteraemia with GNB, comparing success of decontamination of GNB within 14 days, p-value = 0.012, HR = 0.134

| Probability of ICU-acquired bacteraemia or candidemia with: | comparing success versus failure of decontamination of GNB within x days | p-value | HR |
| --- | --- | --- | --- |
| *Staphylococcus aureus* | 3 days | 0.592 | 0.731 |
| *Staphylococcus aureus* | 6 days | 0.458 | 0.581 |
| *Staphylococcus aureus* | 9 days | 0.668 | 0.793 |
| *Staphylococcus aureus* | 12 days | 0.301 | 0.282 |
| *Staphylococcus aureus* | 14 days | 0.266 | 0.256 |
| Streptococci spp. | 3 days | 0.840 | 0.844 |
| Streptococci spp. | 6 days | 0.110 | 5.622 |
| Streptococci spp. | 9 days | 0.999 | 4.28e+08 |
| Streptococci spp. | 12 days | 0.999 | 3.68e+08 |
| Streptococci spp. | 14 days | 0.999 | 3.42e+08 |
| Candida spp. | 3 days | 0.320 | 1.740 |
| Candida spp. | 6 days | 0.332 | 1.987 |
| Candida spp. | 9 days | 0.999 | 4.45e+08 |
| Candida spp. | 12 days | 0.999 | 3.78e+08 |
| Candida spp. | 14 days | 0.999 | 3.63e+08 |
